# Efficacy and safety of PERIOdontal treatment versus usual care for Nonalcoholic liver disease: protocol of the PERION multicenter, two-arm, open-label, randomized trial

**DOI:** 10.1186/s13063-020-4201-y

**Published:** 2020-03-23

**Authors:** Yohei Kamata, Takaomi Kessoku, Tomoko Shimizu, Takashi Kobayashi, Takeo Kurihashi, Satsuki Sato, Syotaro Kuraji, Norio Aoyama, Tomoyuki Iwasaki, Shogo Takashiba, Nobushiro Hamada, Toshiro Kodama, Toshiyuki Tamura, Satoshi Ino, Takuma Higurashi, Masataka Taguri, Takeharu Yamanaka, Masato Yoneda, Haruki Usuda, Koichiro Wada, Atsushi Nakajima, Masato Minabe

**Affiliations:** 1 Department of Highly Advanced Oral Stomatology, Yokohama Clinic, Kanagawa Dental University, 3-31-6 Tsuruya-cho, Kanagawa, Yokohama, Kanagawa 221-0835 Japan; 2 Department of Gastroenterology and Hepatology, Yokohama City University Graduate School of Medicine, 3-9 Fukuura, Kanazawa-ku, Yokohama, 236-0004 Japan; 3 Department of Internal Medicine, Yokohama Clinic, Kanagawa Dental University, 3-31-6 Tsuruya-cho, Kanagawa, Yokohama, Kanagawa 221-0835 Japan; 4 Division of Periodontology, Department of Oral Interdisciplinary Medicine, Graduate School of Dentistry, Kanagawa Dental University, 82 Inaoka-cho, Yokosuka, Kanagawa 238-8580 Japan; 5 Iwasaki Internal Medicine Clinic, 1-1-5 Furu-ruyokohama1F, Kamihoshikawa, Hodogaya-ku Yokohama, Kanagawa 240-0042 Japan; 6 Department of Pathophysiology – Periodontal Science, Okayama University Graduate School of Medicine, Dentistry and Pharmaceutical Sciences, 2-5-1 Shikata-cho, Kita-ku, Okayama, 700-8525 Japan; 7 Division of Microbiology, Department of Oral Science Graduate School of Dentistry, Kanagawa Dental University, 82 Inaoka-cho, Yokosuka, Kanagawa 238-8580 Japan; 8 Department of Implantology and Periodontology, Graduate School of Dentistry, Kanagawa Dental University, 3-31-6 Tsuruya-cho, Kanagawa, Yokohama, Kanagawa 221-0835 Japan; 9 Division of Prosthetic Dentistry, Department of Highly Advanced Stomatology, Graduate School of Dentistry, Kanagawa Dental University, 3-31-6 Tsuruya-cho, Kanagawa, Yokohama, Kanagawa 221-0835 Japan; 10 Department of Biostatistics, Yokohama City University Graduate School of Medicine, 3-9 Fukuura, Kanazawa-ku, Yokohama, 236-0004 Japan; 11 Department of Pharmacology, Shimane University School of Medicine, 89-1 Enya-cho Izumo, Shimane, 693-0581 Japan

**Keywords:** NAFLD, *Porphyromonas gingivalis*, Periodontal treatment, Lipopolysaccharides, Alanine aminotransferase, Immunoglobulin G

## Abstract

**Background:**

We report the first protocol for a multicenter, randomized comparison study to compare the efficacies of periodontal scaling and root-planing treatment against that of tooth-brushing treatment for nonalcoholic fatty liver disease (NAFLD) (PERION: PERIOdontal treatment for NAFLD). Nonalcoholic steatohepatitis (NASH) is an advanced form of NAFLD, which can progress to cirrhosis and hepatocellular carcinoma. Increased endotoxemia is associated with the progression of NAFLD. Periodontal bacteria possess endotoxins; *Porphyromonas gingivalis* is well-known as a major pathogenic bacterium in periodontitis, and serum antibody levels for *P. gingivalis* are high in patients with periodontitis. Several reports have indicated that *P. gingivalis* is related to NAFLD. This study aims to investigate the effect of periodontal treatment for liver damage, *P. gingivalis* infection, and endotoxemia on patients with NAFLD.

**Methods:**

We will include adult patients (20–85 years old) with NAFLD, alanine aminotransferase (ALT) ≥ 40 IU/L, and equivalent steatosis grade ≥ 1 (target sample size, *n* = 40 patients; planned number of patients with outcome data, *n* = 32). Participants will be randomly assigned to one of two groups: a scaling and root-planing group or tooth-brushing as the usual group. The primary outcome will be the change in ALT levels from baseline to 12 weeks; the key secondary outcome will be the change in the serum immunoglobulin G (IgG) antibody titer for *P. gingivalis* at 12 weeks.

**Discussion:**

This study should determine whether periodontal treatment decreases liver damage, *P. gingivalis* infection, and endotoxemia in patients with NAFLD.

**Trial registration:**

University Hospital Medical Information Network (UMIN) Clinical Trials Registry, ID: UMIN000022079.

## Background

The broad spectrum of fatty liver diseases in individuals who consume little-to-no alcohol is called nonalcoholic fatty liver disease (NAFLD) and includes nonalcoholic steatohepatitis (NASH). NASH is an increasingly common cause of chronic liver disease worldwide and is associated with increased liver-related mortality and hepatocellular carcinoma [1–3]. NASH progresses to cirrhosis in 15–20% of the affected individuals and is a rising indication for liver transplantation [4]. However, approved therapies for NASH have not yet been established; therefore, preventive therapies to inhibit the progression of fatty liver disease to NASH are required.

Periodontal disease is an infectious disease of the gums and tissues surrounding the teeth and causes tooth loss resulting from the destruction of tooth-supporting tissues. The incidence rate of periodontitis is >  47% in adults in the USA [5]. More than 700 bacterial species or phylotypes have been detected in the oral cavity [6]. Some species/complexes are closely associated with advanced periodontal lesions, such as *Porphyromonas gingivalis*, *Treponema denticola*, *Tannerella forsythia*, *Prevotella intermedia*, *Fusobacterium nucleatum*, and *Aggregatibacter actinomycetemcomitans* [7, 8]. Among them, *P. gingivalis*, a gram-negative anerobic bacterium, is the major etiologic agent that contributes to periodontal disease progression and bone and tissue destruction [9, 10]. The lipopolysaccharide (LPS) cell-wall component of *P. gingivalis* is one of the virulence factors that trigger a wide range of host responses, including the production of pro-inflammatory cytokines, anti-inflammatory cytokines, and chemokines [11]. These cytokines and inflammatory mediators play important roles in the progression of periodontitis at the stage where host immune and inflammatory responses lead to the destruction of periodontal tissue under the influence of multiple behavioral, environmental, and genetic factors [12].

**Fig. 1:**
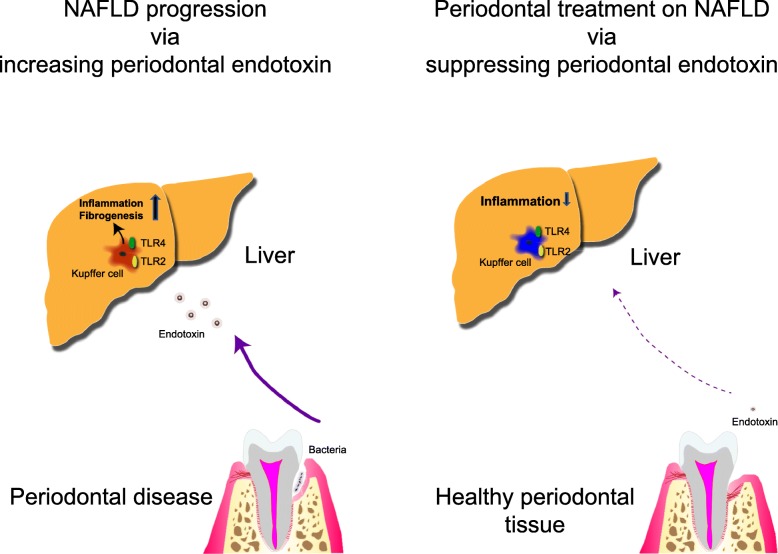
Schematic overview of periodontal treatment of NAFLD suppressing periodontal endotoxin. *NAFLD* nonalcoholic fatty liver disease, *TLR2/TLR4* toll-like receptors 2 and 4

Recently, several studies have reported the relationship between NAFLD and periodontal disease [13, 14]. Yoneda et al. [15] reported that the detection frequency of *P. gingivalis* in the saliva of patients with NAFLD and patients with NASH was significantly higher than that in non-NAFLD control subjects. Moreover, they presented preliminary evidence to suggest that nonsurgical periodontal treatments in 10 patients with NAFLD for 3 months ameliorated the liver function parameters, such as the serum levels of aspartate aminotransferase (AST) and alanine aminotransferase (ALT). Consequently, it is thought that infection with a periodontal pathogen, mainly *P. gingivalis*, is associated with fibrosis severity in patients with NAFLD and that the prevention and elimination of *P. gingivalis* infection by periodontal treatment may have a beneficial effect on the management of NASH (Fig. 1).

Therefore, we hypothesized that the elimination of oral infection, including *P. gingivalis* infection, by periodontal treatment in patients with NAFLD would ameliorate NAFLD-related clinical markers. We performed a clinical study to confirm the preliminary finding under collaborative medical and dental care.

Thus, we have devised a prospective, multicenter, randomized comparison trial to evaluate periodontal treatment as a candidate for NAFLD treatment. This is the first protocol for a randomized comparison trial for periodontal treatment against NAFLD in humans.

## Methods

### Design

**Fig. 2:**
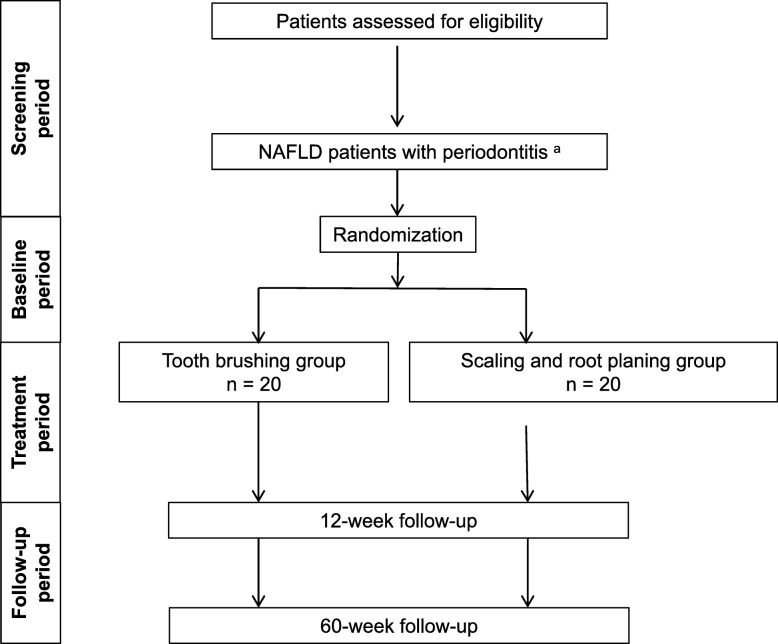
Study design for PERION. Planned sample size, *N* = 32; enrolled, *N* = 40. PERION, PERIOdontal treatment for NAFLD, *NAFLD* nonalcoholic fatty liver disease

The PERION trial is designed as a prospective, multicenter, two-arm, randomized comparison study to test the efficacy of the 12-week scaling and root-planing group versus the tooth-brushing group in NAFLD with moderate periodontitis. The study will recruit 40 adults and evaluate the efficacy and safety of periodontal treatment for 60 weeks, with the primary endpoint at 12 weeks. The study design is shown in Fig. 2.

### Recruitment process and allocation

The PERION trial patient population will be derived from the Kanagawa Dental University Yokohama Clinic, Kanagawa Dental University, Iwasaki Internal Medicine Clinic, and the Yokohama City University Hospital Cohort. The randomized allocation will be conducted at Yokohama City University. Eligible patients will be screened by the principal and sub-investigator (gastroenterologists and periodontists). Patient recruitment will be performed 8 h a day, 5 days a week.

### Endpoint detection

**Fig. 3:**
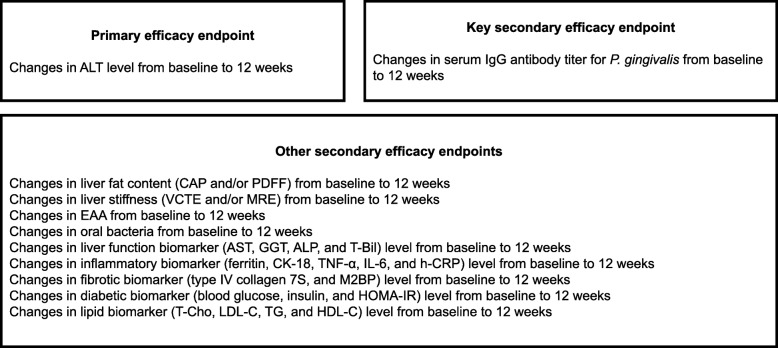
Efficacy endpoints for PERION. *ALP* alkaline phosphatase, *ALT* alanine aminotransferase, *AST* aspartate aminotransferase, *BMI* body mass index, *CAP* controlled attenuation parameter, *CK-18* cytokeratin 18, *CRP* C-reactive protein, *EAA* endotoxin activity assay, *GGT* γ-glutamyltransferase, *h-CRP* high-sensitivity C-reactive protein, *HDL-C* high-density lipoprotein-cholesterol, *HOMA-IR* homeostasis model assessment of insulin resistance, *HRQOL* health-related quality of life, *IL-6* interleukin-6, *LDL-C* low-density lipoprotein-cholesterol, *M2BP* Mac-2 binding protein, *MRE* magnetic resonance elastography, *NAFLD* nonalcoholic fatty liver disease, *PERION* PERIOdontal treatment for NAFLD, *PDFF* proton density fat fraction, *SF-8* short form-8, *QOL* quality of life, *T-Bil* total bilirubin, *T-Cho* total cholesterol, *TG* triglycerides, *TNF-α* tumor necrosis factor-α, *VCTE* vibration-controlled transient elastography

In the short-term studies (Phases I and IIa), aimed primarily at detecting wasted signals to make direct decisions on further development, a sustained improvement in AST and ALT levels will be useful as the endpoint of PERION (Fig. 3) [16]. While the use of ALT as a surrogate marker for NAFLD is controversial, studies have shown that ALT reduction is associated with reduced hepatocyte damage and liver inflammation [17], but not steatosis [18]. Because there are no other commonly established noninvasive biomarkers for use in the NAFLD/NASH clinical trial, ALT reduction was selected as the primary endpoint of this trial. To assist the primary endpoint, several secondary endpoints were selected to assess the pathogenesis of NAFLD using noninvasive methods (Fig. 3). Therefore, the primary endpoint of PERION is set as a change in ALT levels from baseline after 12 weeks of intervention. Also, the PERION study will evaluate periodontal treatment as a candidate for the first treatment to improve NAFLD pathogenesis via decreasing *P. gingivalis* count. Therefore, the key secondary endpoint is the serum immunoglobulin G (IgG) antibody titer of *P. gingivalis* [19].

### Periodontal disease examination

The subject will be examined to assess the amount of periodontal disease bacteria (*P. gingivalis,* etc.), degree of infection, and periodontal disease severity. All examinations will be performed by two periodontal disease specialists enrolled at Kanagawa Dental University Hospital and Kanagawa Dental University Yokohama Clinic. The amount of *P. gingivalis* in saliva is measured by quantitative polymerase chain reaction (qPCR), and the infection level of periodontal disease is examined using a serum IgG antibody titer test for *P. gingivalis* FDC381 by enzyme-linked immunosorbent assay (ELISA). The severity of periodontal disease will be examined by probing depths, clinical attachment levels, gingival bleedings on probing (BOP) at six sites per tooth using a calibrated periodontal probe, and the stability of the teeth.

### Periodontal treatment

The primary purpose of nonsurgical periodontal treatment is to control periodontal infection of microorganisms by removing bacterial biofilm, calculus, and toxins from the root surface. According to a review of the scientific literature, mechanical nonsurgical periodontal treatment significantly reduces the inflammation level and the probing pocket depth and increases the clinical attachment level [20]. The successful treatment of plaque-induced periodontitis will recover periodontal health, but gingival recession often occurs. At first, instruction on correct brushing of the teeth will be given to patients with periodontal disease (tooth-brushing group). Then, the removal of supra- and sub-gingival bacterial plaque/biofilm and calculus by periodontal scaling and root-planing will be performed. Quadrant/sextant-wise instrumentation (conventional staged debridement, CSD) will be performed (scaling and root-planing group).

### NAFLD treatment

We will provide standard lifestyle modification recommendations that each site can provide to the patients at the time that the informed consent document is signed. We will recommend a hypocaloric diet (daily calorie reduction of 500–1000 kcal) with reduced consumption of processed carbohydrates and fructose-containing beverages and will recommend the performance of moderate-intensity exercise for 30–45 min three to four times/week. This diet and exercise therapy will be performed without changing the prescription.

### Sample size determination

Our previous pilot study showed that nonsurgical periodontal treatments on 10 NAFLD patients for 3 months ameliorated the liver function parameters, such as the serum levels of AST and ALT [15]. It showed a mean ALT change of − 25.0 (IU/L), with a standard deviation of 25. On the basis of these data, the sample size is determined to guarantee the power of the analysis of variance *F* test. Assuming the mean changes in ALT in the no-treatment group and active treatment groups to be 0 and − 25, respectively, with a common standard deviation of 25, the required number of patients per group with a power of 80% and a two-sided significance level of 5% was calculated to be 16. We aim to recruit a total of 40 patients (scaling and root-planing treatment, 20, tooth-brushing treatment, 20) to compensate for the dropout patients.

### Eligibility criteria

Patients with NAFLD who will be recruited in this study must satisfy the inclusion and exclusion criteria presented in Tables 1 and 2. Fatty liver, steatosis grade and fibrosis stage will be assessed using noninvasive methods (ultrasound, vibration-controlled transient elastography (VCTE), and magnetic resonance imaging (MRI)).

**Table 1:** Efficacy and safety of PERIOdontal treatment versus usual care for Nonalcoholic liver disease (PERION) inclusion criteria

Criteria type	Description of inclusion criteria
Sex and age	Men and women: 20–85 years of age
Diet and exercise therapy	Patients with NAFLD who did not respond to 3-month diet and exercise therapy
ALT levels	Patients with an ALT level of > 40 IU/L at the start of this study
Fatty liver	Patients with a diagnosis of fatty liver based on abdominal ultrasonography^a^
Steatosis grade	Patients with the equivalent of steatosis grade ≥ 1 on CAP (using FibroScan) and/or PDFF (using MRI)^b^
Fibrosis stage	Patients with the equivalent of fibrosis stage < 4 on TE (FibroScan) and/or MRE^c^
Alcohol consumption	Patients with no habitual alcohol consumption (i.e., consumption of ethanol > 30 g/day in men and > 20 g/day in women)
Periodontitis	Patients with chronic moderate periodontitis (holding rate of periodontal pocket depth of > 4 mm is > 10 sites)
Other	Patients who can provide written consent to participate in this research in person, follow instructions during participation in this research, undergo protocol-specified physical examination and other examinations, and report symptoms or events

*ALT* alanine aminotransferase, *CAP* controlled attenuation parameter, *MRE* magnetic resonance elastography, *MRI* magnetic resonance imaging, *NAFLD* nonalcoholic fatty liver disease, *PERION* PERIOdontal treatment for NAFLD, *PDFF* proton density fat fraction, *VCTE* vibration-controlled transient elastography. ^a^Criteria of fatty liver, as defined by the existence of hepatorenal echo contrast. ^b^Defined by CAP ≥ 236 dB/m and/or PDFF ≥ 5.2%. ^c^Defined by VCTE < 14 kPa and/or MRE < 6.7 kPa

**Table 2:** Efficacy and safety of PERIOdontal treatment versus usual care for Nonalcoholic liver disease (PERION) exclusion criteria

Criteria type	Description of exclusion criteria
Liver comorbidity	Patients with any other concurrent liver disease, such as hepatitis C, hepatitis B, or autoimmune hepatitis
	Patients with drug-induced symptomatic NAFLD
Other comorbidity	Patients with concurrent or past history of any serious cardiac, vascular, hematological, respiratory, hepatic, renal, gastrointestinal, or neuropsychiatric disease
	Patients with a history of abdominal or gastrointestinal surgery, except appendicitis
Medication	Patients with any change to their orally administered medications within 3 months before informed consent
	Patients with diabetes mellitus being treated with insulin injections
Other	Patients who have participated in any other clinical study and received study treatment within 1 month before the start of this research (counted from the first day of study medication)
	Breastfeeding women or women with possible pregnancy
	Other patients who are inappropriate as participants in this research in the opinion of the principal investigator, etc.

*NAFLD* nonalcoholic fatty liver disease, PERION PERIOdontal treatment for NAFLD

### Randomization

Before providing informed consent, patients will undergo a screening test as a first step to determine whether they meet the inclusion criteria for the study and none of the exclusion criteria. For patients determined to be eligible, the principal investigator or co-investigator will complete a Patient Enrollment Form with the necessary information, which is sent by fax or e-mail to the Patient Enrollment Center (Yokohama City University). The Patient Enrollment Center will then confirm the eligibility of the patient based on the enrollment form, enroll and randomize the patient, and notify the principal investigator or co-investigator of the Patient ID number and the allocation number via fax or e-mail. After an eligibility check, patients will be randomly assigned to receive periodontal treatment or no treatment at the Central Registration Center by a computer program, using a stratified block randomization method, adjusting for age (≥ 65/< 65 years) and sex (male/female). Therefore, the patient assignment is concealed from the investigator.

### Study procedures

The schedule of assessments for the study procedures is summarized in Table 3.

**Table 3:** Objectives and procedures of the Efficacy and safety of PERIOdontal treatment versus usual care for Nonalcoholic liver disease (PERION) study

	Study time point (weeks)					
	Screening period	Treatment period				Follow-up period
Study objectives	− 4	0	4	8	12	60
**Primary objective**						
Change in ALT levels from baseline	○	○			○	○
**Key secondary objective**						
Change in serum IgG antibody titer for *P. gingivalis*	○				○	○
**Other secondary objectives**						
Change in blood endotoxin activity by EAA	○	○	○	○	○	○
Change in liver fat content using CAP and MRI-PDFF	○	○			○	
Change in liver stiffness using VCTE and MRE	○	○			○	
Change in oral bacterial counts using NGS and qPCR	○	○			○	
Change in blood parameters for liver function (AST, GTT, ALP, and T-Bil)	○	○	○	○	○	○
Change in blood lipid parameters (T-Cho, LDL-C, TG, and HDL-C)	○	○	○	○	○	○
Change in blood parameters related with inflammation in NAFLD (ferritin, CK-18, TNF-α, IL-6, and h-CRP)		○			○	
Change in blood parameters related with fibrotic marker in NAFLD (type IV collagen 7S)		○			○	
Change in blood diabetic factors (blood glucose, insulin, and HOMA-IR)		○	○	○	○	○
Change in BMI	○	○	○	○	○	○
Assessment of periodontal treatment safety		○	○	○	○	○
Dropout ratio in each group		○	○	○	○	○
**Tertiary objectives**						
Change in blood parameters for renal function (BUN, Cr, eGFR)	○	○	○	○	○	○
Change in HRQOL using SF-8™	○	○			○	○

All objectives will be compared between the periodontal scaling and root-planing treatment group and the tooth-brushing treatment group. *ALP* alkaline phosphatase, *ALT* alanine transaminase, *AST* aspartate transaminase, *BMI* body mass index, *BUN* blood urea nitrogen, *CAP* controlled attenuation parameter, *CK-18* cytokeratin 18, *Cr* creatinine, *EAA* endotoxin activity assay, *eGFR* estimated glomerular filtration rate, *FBS* fasting blood sugar, *GGT* γ-glutamyltransferase, *h-CRP* high-sensitivity C-reactive protein, *HDL-C* high-density lipoprotein-cholesterol, *HOMA-IR* homeostasis model assessment of insulin resistance, *HRQOL* health-related quality of life, *IL-6* interleukin-6, *LDL-C* low-density lipoprotein-cholesterol, *MRE* magnetic resonance elastography, *MRI* magnetic resonance imaging, *NAFLD* nonalcoholic fatty liver disease, *NGS* next-generation sequencer; PERION, PERIOdontal treatment for NAFLD, *PDFF* proton density fat fraction, *qPCR* quantitative polymerase chain reaction, *SF-8* short form-8, *T-Bil* total bilirubin, *T-Cho* total cholesterol, *TG* triglycerides, *TNF-α* tumor necrosis factor-α

#### Observation of adverse events

All adverse events (AEs) during the study will be recorded with regard to the following: date of onset and date of completion (if applicable), severity of AEs, investigator’s view on the relationship to periodontal treatment, information on actions taken, information on the treatment of the AE, the cause of the event (if known), and the solution or outcome. AEs classified as serious will be recorded in the serious adverse event reporting tool and reported to the sponsor. AE intensities are graded according to the National Cancer Institute’s Adverse Event Common Terminology (NCI CTCAE) version 4.03, including the AE-intensity classifications shown in Table 4.

**Table 4:** Classifications of adverse event (AE) intensity

Grade	Description
Grade 1 (mild)	Asymptomatic or mild symptoms; clinical or diagnostic observations only; intervention not indicated
Grade 2 (moderate)	Minimal, local, or noninvasive intervention indicated; limiting age-appropriate instrumental ADL
Grade 3 (severe)	Medically significant but not immediately life-threatening; hospitalization or prolongation of hospitalization indicated; disabling; limiting self-care ADL
Grade 4 (life-threatening)	Life-threatening consequences; urgent intervention indicated
Grade 5 (death)	Death related to AE

*ADL* activities of daily living, *AE* adverse event

#### Additional study procedures

Patients must be fasted for at least 8 h prior to the visit. The study visits will be at 0, 4, 8, 12, and 60 weeks.

#### Criteria for discontinuation of study treatment

The study must be discontinued under the following conditions: drug-induced liver injury, unacceptable toxicity, acute viral hepatitis B and C, autoimmune or alcoholic hepatitis, hypoxic or ischemic liver injury, biliary tract disease, or pregnancy.

### Outcome measures

#### Evaluation of efficacy

The efficacy endpoints for this study are shown in Fig. 3.

#### Evaluation of safety

The safety and tolerability of periodontal treatment will be evaluated over the 60 weeks of treatment in NAFLD patients with periodontitis. This will include the evaluation of AEs, clinical laboratory tests, physical examination, and vital signs. The clinical laboratory tests will include liver and fasting metabolic parameters. Liver parameters will include alkaline phosphatase (ALP), AST, ALT, total bilirubin (T-Bil), and γ-glutamyltransferase (GGT).

### Statistical analyses

The Full Analysis Set (FAS) will be the primary analysis set for efficacy. We define FAS to include any subjects who receive any amount of the study medication without a lack of information on the primary endpoint (complete case analysis). For the blood liver function test using ALT, the summary statistics (mean, standard deviation) will be calculated at baseline and 12 weeks. Details of the statistical design will be described in the Statistical Analysis Plan. In this study, statistical analysis will be performed mainly for the following items. For the primary analysis, (ALT after treatment – ALT at baseline) will be sought for each subject and the corresponding Wilcoxon test will be performed. The significance level will be 5% on both sides. In addition to *p* values, we will provide point estimates with 95% confidence intervals. As a sensitivity analysis, the two groups would be compared using the analysis of covariance using the primary endpoint as an outcome, adjusting for ALT at baseline as a covariate. For the key secondary endpoint, *P. gingivalis* IgG antibody titer in the blood (values after treatment – values at baseline) will be sought for each subject and the paired Wilcoxon test will be performed. Subgroup analysis will be conducted. Stratified analysis will be performed using *P. gingivalis* bacterial content, proton density fat fraction (PDFF), magnetic resonance elastography (MRE), and periodontal pocket as indices. Stratified analysis will be performed using above/below the median of *P. gingivalis* bacterial content, endotoxin, PDFF, MRE, and periodontal pocket depth. We hypotheses a larger reduction in ALT with the intervention in the patient group with high *P. gingivalis* bacterial content, high endotoxin levels, high PDFF, low MRE, and deep periodontal pockets at baseline. Therefore, we conducted multivariable linear regression with difference in ALT between groups as dependent variable and at least the treatment group, the respective subgroup variable (categorized), and the interaction term (treatment group×subgroup variable) as independent variables. The significance level will be 5% on both sides. In addition to p values, we will provide point estimates with 95% confidence intervals. As a sensitivity analysis, the two groups will be compared using analysis of covariance using the key secondary endpoint as an outcome adjusting for *P. gingivalis* IgG antibody titer at baseline as a covariate. As the safety analysis, the incidence and severity of AEs and reactions will be calculated.

### Trial Steering Committee and Data Monitoring Committee

The Trial Operation Committee is integrated and consists of three persons appointed by independent clinical and basic investigators (a general internist in primary care, a palliative care specialist and a statistician from Yokohama City University School of Medicine). They provide overall supervision and ensure that all registered trials. These investigators are anonymous and randomly selected. The Independent Data Monitoring Committee will be established with two persons from the Department of Biostatistics, Yokohama City University School of Medicine. The management team will observe progress and data monthly by phone call, mail, and/or web-conferencing. If the Monitoring Committee decides that on-site monitoring is necessary, monitoring members will visit the site for face-to-face monitoring.

## Discussion

This is the first study proposed to explore the effect of periodontal treatment on NAFLD patients with periodontitis. The primary endpoint used in previous studies was the liver histology, which was evaluated by a liver biopsy (Pivens, Flint, and Golden study) [21–24]. Liver histology endpoints, such as the complete resolution of NASH, are considered surrogates for preventing cirrhosis (i.e., they are thought to predict a clinical benefit but are not direct measurements of it). However, due to increased cost, possible risk, inter- and intra-observer bias, and healthcare resource utilization, an invasive liver biopsy is poorly suited as a diagnostic test for such a prevalent condition [25]. Furthermore, the histological lesions of NASH are unevenly distributed throughout the liver parenchyma; therefore, liver biopsy sampling error can result in substantial stratification and staging inaccuracies [26]. Currently, the noninvasive methods used to assess NASH progression are not robust enough to replace liver biopsy. We considered that it is important to perform noninvasive, safe, low-cost, and short-term clinical trials as proof-of-concept studies. Many studies reported that the steatosis grade can be evaluated using controlled attenuation parameter (CAP) [27–32], which is based on the properties of ultrasonic signals acquired by VCTE, and MRI-PDFF [33, 34], which is an MRI-based method for quantitatively assessing hepatic steatosis and is available from several manufacturers of MRI scanners. Moreover, VCTE and MRE have superior diagnostic ability to evaluate steatosis and fibrosis in patients with NAFLD [35, 36]. Therefore, we consider that noninvasive evaluation of NAFLD pathogenesis using VCTE and MRI could replace invasive methods, such as liver biopsy, as a proof-of-concept study. Therefore, noninvasive methods, such as VCTE and MRI, to assess NASH/NAFLD progression, were included as secondary endpoints to compare against liver biopsy.

Clinical research in Japanese university students has suggested that having periodontal disease in young men was significantly associated with an increased level of ALT [37]. In addition, the incidence of periodontal disease in healthy Japanese women was reported to be significantly increased with elevated serum levels of AST, ALT, and cholinesterase [38]. Furthermore, an observational study with annual workplace health check-ups at a company in Japan reported an association between periodontal condition and the combination of elevated ALT and metabolic syndrome (MetS) in men [39]. Besides, it has been suggested that more severe periodontal disease is associated with increased serum levels of GGT, a liver biochemical parameter, in Japanese adults with no alcohol-drinking habits [40]. In an in-vivo mouse study, it was demonstrated that areas of fibrosis with proliferation of hepatic stellate cells and collagen formation were observed in mice with *P. gingivalis* infection fed on a high-fat diet. In addition, in steatotic hepatocytes, the expression of toll-like receptor 2 (TLR2), one of the *P. gingivalis*-LPS receptors, was upregulated. *P. gingivalis*-LPS further increased messenger ribonucleic acid (mRNA) levels of palmitate-induced inflammasome and proinflammatory cytokines in steatotic hepatocytes [41]. That is to say, the dental infection of *P. gingivalis* exacerbated the pathological progression of NASH from simple steatohepatitis to steatohepatitis with fibrosis through the upregulation of the *P. gingivalis*-LPS-TLR2 pathway and activation of inflammasomes. Recently, evidence in mice has shown that disturbance of the gut microbiota composition by orally derived periodontopathic bacteria, such as *P. gingivalis*, may be a causal mechanism linking periodontitis and systemic disease including NAFLD [42–44].

In conclusion, this study should determine whether periodontal treatment to decrease endotoxin levels and treat *P. gingivalis* infection improves the disease status of patients with NAFLD. The PERION study is the first randomized controlled study suppressing hyper-endotoxemia in NAFLD with periodontitis. This study should allow the assessment of the efficacy and safety of periodontal treatment in a larger population of NAFLD patients with periodontitis.

### Strengths

To our knowledge, no direct comparison has been made between randomized controlled groups of patients with NAFLD with periodontitis. The strength of this study is that non-invasive MRI-PDFF will be used to assess changes in hepatic fat mass, as opposed to liver biopsy. Importantly, improved laboratory tests for periodontal disease and *P. gingivalis* removal may correlate with improved liver fat mass and liver function.

### Limitations

There are several limitations in our study. First, the small sample size and short treatment period (3 months) limit our findings. Second, it is important to differentiate NASH in NAFLD patients; however, at present, the gold standard for such differentiation is liver-tissue diagnosis by liver biopsy. In this study, NAFLD patients would not have undergone liver biopsy. Third, our protocol is open-label. Fourth, the specified primary and key secondary outcomes are only surrogate outcomes. The extent of decrease in ALT or IgG antibody titers will translate into improved quality of life (QOL) or any other more patient-relevant outcomes of NAFLD patients will not be determined in the present trial.

### Trial status

Recruitment of participants begun in August 2015 and will be open until March 2020.

Current approved protocol: Version 1.3, 24 January 2018.

## Data Availability

Not applicable

## Abbreviations

ALP: Alkaline phosphatase
ALT: Alanine aminotransferase
AST: Aspartate aminotransferase
BMI: Body mass index
CAP: Controlled attenuation parameter
CK-18: Cytokeratin 18
CRP: C-reactive protein
EAA: Endotoxin activity assay
eGFR: Estimated glomerular filtration rate
GGT: γ-glutamyltransferase
h-CRP: High-sensitivity C-reactive protein
HDL-C: High-density lipoprotein-cholesterol
HOMA-IR: Homeostasis model assessment of insulin resistance
HRQOL: Health-related quality of life
IEC: Independent Ethics Committee
IL-6: Interleukin-6
IRB: Institutional Review Board
LDL-C: Low-density lipoprotein-cholesterol
LPS: Lipopolysaccharide
LSM: Liver stiffness measurement
MRE: Magnetic resonance elastography
MRI: Magnetic resonance imaging
NAFLD: Nonalcoholic fatty liver disease
NASH: Nonalcoholic steatohepatitis
PDFF: Proton density fat fraction
QOL: Quality of life
qPCR: Quantitative polymerase chain reaction
SF-8: Short form-8
T-Bil: Total bilirubin
T-Cho: Total cholesterol
TG: Triglyceride
TLR2: Toll-like receptor 2
TLR4: Toll-like receptor 4
TNF-α: Tumor necrosis factor-α
VCTE: Vibration-controlled transient elastography
